# Detecting Motor Impairment in Early Parkinson’s Disease via Natural Typing Interaction With Keyboards: Validation of the neuroQWERTY Approach in an Uncontrolled At-Home Setting

**DOI:** 10.2196/jmir.9462

**Published:** 2018-03-26

**Authors:** Teresa Arroyo-Gallego, María J Ledesma-Carbayo, Ian Butterworth, Michele Matarazzo, Paloma Montero-Escribano, Verónica Puertas-Martín, Martha L Gray, Luca Giancardo, Álvaro Sánchez-Ferro

**Affiliations:** ^1^ Institute for Medical Engineering and Science Massachusetts Institute of Technology Cambridge, MA United States; ^2^ Biomedical Image Technologies Universidad Politécnica de Madrid Madrid Spain; ^3^ Biomedical Research Networking Centre thematic area of Bioengineering, Biomaterials and Nanomedicine Madrid Spain; ^4^ nQ Medical Inc Cambridge, MA United States; ^5^ Research Laboratory of Electronics Massachusetts Institute of Technology Cambridge, MA United States; ^6^ Centro Integral de Neurociencias A.C. Hospital Universitario HM Puerta del Sur Móstoles Spain; ^7^ Neurology Department Instituto de Investigación del Hospital 12 de Octubre Madrid Spain; ^8^ Enfermedades Neurodegenerativas Centro de Investigación Biomédica en Red Madrid Spain; ^9^ Pacific Parkinson’s Research Centre The University of British Columbia Vancouver, BC Canada; ^10^ Movement Disorders Unit Hospital Clínico San Carlos Madrid Spain; ^11^ Center for Precision Health School of Biomedical Informatics The University of Texas Health Science Center at Houston Houston, TX United States; ^12^ Medical School CEU-San Pablo University Madrid Spain

**Keywords:** eHealth, machine learning, telemedicine

## Abstract

**Background:**

Parkinson’s disease (PD) is the second most prevalent neurodegenerative disease and one of the most common forms of movement disorder. Although there is no known cure for PD, existing therapies can provide effective symptomatic relief. However, optimal titration is crucial to avoid adverse effects. Today, decision making for PD management is challenging because it relies on subjective clinical evaluations that require a visit to the clinic. This challenge has motivated recent research initiatives to develop tools that can be used by nonspecialists to assess psychomotor impairment. Among these emerging solutions, we recently reported the neuroQWERTY index, a new digital marker able to detect motor impairment in an early PD cohort through the analysis of the key press and release timing data collected during a controlled in-clinic typing task.

**Objective:**

The aim of this study was to extend the in-clinic implementation to an at-home implementation by validating the applicability of the neuroQWERTY approach in an uncontrolled at-home setting, using the typing data from subjects’ natural interaction with their laptop to enable remote and unobtrusive assessment of PD signs.

**Methods:**

We implemented the data-collection platform and software to enable access and storage of the typing data generated by users while using their computer at home. We recruited a total of 60 participants; of these participants 52 (25 people with Parkinson’s and 27 healthy controls) provided enough data to complete the analysis. Finally, to evaluate whether our in-clinic-built algorithm could be used in an uncontrolled at-home setting, we compared its performance on the data collected during the controlled typing task in the clinic and the results of our method using the data passively collected at home.

**Results:**

Despite the randomness and sparsity introduced by the uncontrolled setting, our algorithm performed nearly as well in the at-home data (area under the receiver operating characteristic curve [AUC] of 0.76 and sensitivity/specificity of 0.73/0.69) as it did when used to evaluate the in-clinic data (AUC 0.83 and sensitivity/specificity of 0.77/0.72). Moreover, the keystroke metrics presented a strong correlation between the 2 typing settings, which suggests a minimal influence of the in-clinic typing task in users’ normal typing.

**Conclusions:**

The finding that an algorithm trained on data from an in-clinic setting has comparable performance with that tested on data collected through naturalistic at-home computer use reinforces the hypothesis that subtle differences in motor function can be detected from typing behavior. This work represents another step toward an objective, user-convenient, and quasi-continuous monitoring tool for PD.

## Introduction

### Background

Parkinson’s disease (PD) is the second most prevalent neurodegenerative disorder affecting 0.3% of the general population and about 1% in people over 60 years [1]. Today, PD diagnosis and management rely on the clinical judgment of neurologists to detect and evaluate the severity of motor and nonmotor manifestations of the disease [2]. The Unified Parkinson’s Disease Rating Scale (UPDRS) is the most widely used method to assess the longitudinal course of PD [3,4]. The UPDRS score comprises 4 subscales, including the clinician-scored motor section (UPDRS-III) that provides a comprehensive evaluation of PD motor degeneration through the evaluation of the observed performance in a series of specific motor tasks [5]. Administered typically by a movement disorder specialist, this scale requires significant training to minimize rater bias [6]. The need of a trained specialist intrinsically limits the frequency at which disease status and progression can be assessed to a number of on-site clinical evaluations, usually every 2 to 6 months [7].

There has been substantial interest in the last decade to develop tools that can assess motor function in PD without the need for specialist training or even on-site administration [8]. Such tools could complement the current standard, introducing the potential for greater screening opportunities or an increased assessment frequency for tracking changes. A variety of technological approaches have been designed for use in the clinic, such as finger-tapping that introduces a series of standardized finger-movement tasks that provide quantitative measurements of motor impairment [9]. Additionally, out-of-clinic approaches have been trialed, such as the mPower initiative [10], a smartphone-based activity tracker that collects longitudinal data from a series of tasks and surveys specifically designed to evaluate the progression of PD symptoms.

### Objective

Our project focuses on the analysis of finger-keyboard interaction to assess psychomotor impairment. We have previously shown that we can extract information relevant to users’ psychomotor status by timing the keystroke events during a typing task using a mechanical keyboard [11]. In Giancardo et al [12], we showed that it was possible to derive an early-PD phenotype based on a metric derived from the typing data acquired in a controlled clinical environment. Subjects were asked to transcribe a randomly selected folktale using a word processor on a standard 15-inch laptop during a 15-min timed routine.

The widespread use of personal electronics has placed typing among the activities of our daily routine. This enables the possibility of leveraging the data from users’ natural interaction with their devices to apply our method in an unobtrusive manner. From a data-collection standpoint, it is straightforward to extend our technology to collect timing information in a naturalistic ecologically valid scenario (eg, home). However, from the standpoint of data analysis, passive monitoring poses interesting challenges that could affect the application of our method to evaluate at-home natural typing. From a data-sampling perspective, typing happens in unpredictable bursts that introduce a high degree of sparsity in the resulting typing signals. The various contexts in which the typing data are generated at-home may also add difficulty in contrast with the controlled copy task performed in the clinic. Finally, hardware heterogeneity introduces a potential confounder in the at-home setting, which we were able to control in our in-clinic setting using a single machine approach.

In this paper, we present the results of the validation of our in-clinic-built algorithm to detect PD typing patterns in an uncontrolled at-home setting. We implemented a data-collection platform that allowed us to passively collect the typing information from subjects’ daily interaction with their laptop. Our algorithm performed well prospectively on a controlled typing study conducted in the clinic. Here, we examine whether the same algorithm performs well on the typing data collected at home and evaluate the influence of the in-clinic typing task in subjects’ normal typing behaviors.

## Methods

### Study

The results presented in this work analyze the baseline data collected as part of a 6-month longitudinal PD drug-responsiveness study (NCT02522065). All the experimental protocols were approved by the Massachusetts Institute of Technology, USA (Committee on the Use of Humans as Experimental Subjects approval no. 1412006804), HM Hospitales, Spain (No. 15.05.796-GHM), Hospital 12 de Octubre, Spain (No. CEIC:14/090), and Hospital Clínico San Carlos, Spain (No.14/136-E). All subjects provided informed consent before study enrollment. The recruitment and experimental procedures were carried out following the relevant institutional guidelines.

The study cohort consisted of 60 subjects, 30 people with recently diagnosed Parkinson’s (PwP) and 30 healthy controls. Only subjects who self-reported at least 30 min of daily laptop use were considered for the study. The exclusion criteria included cognitive impairment, upper limb functional limitation, sleep disorders, and use of antipsychotics or sedative drugs. At the moment of enrollment, 6 PwP were on rasagiline while the remaining 24 were completely drug naïve. Notably, unlike levodopa or dopamine agonists, rasagiline is a compound that has a little impact on motor performance; so, for the purposes of this study related to motor performance, we considered patients on rasagiline to be similar to the PwP who had not yet started medication. They maintained their baseline medication status (ie, no drug or continued rasagiline) for a period of time after the enrollment visit.

The enrolled participants underwent an initial baseline assessment in the clinic that included clinical evaluation, an in-clinic controlled typing test, and the technical setup to enable at-home monitoring. The medical examination included a UPDRS-III-based evaluation carried out by movement disorder specialists. For the in-clinic typing test, the participants were asked to transcribe an unstandardized sample text on a standard word processor during 15 min. To emulate natural interaction with the device, subjects were asked to type as they would normally do at home. A standard machine was used in the in-clinic setting, specifically, a Lenovo G50-70 i3-4005U with 4GB of memory and a 15-inch screen running Manjaro Linux operative system. While undertaking the test, the data-collection software ran in the background. Once the task was completed, the typing data were sent to our database server. As part of the baseline visit workflow, the data-collection software was installed on participants’ personal laptop to enable at-home remote monitoring. If they shared their computer, we provided them with a laptop with preinstalled software. Subjects were encouraged to enter into the routine of typing an email or a document for at least 15 min per day but otherwise use the computer as they would do normally.

Once enrolled in the study, PwP subjects kept their baseline medication status for about a week. This baseline period allowed an unbiased comparison between the in-clinic and at-home conditions on the assessment of our method. Due to the naturalistic design, there was some variability in the time between the initial visit and the date the new therapy was started (ie, some variability in the duration of the baseline period). This period ranged from 0 to 63 days. For the data reported here, we used a 7-day baseline period, unless there was a medication change within that timeframe, in which case we used the actual baseline period. For the control group, the baseline period was defined as the 7-day period since the date they first logged in to the neuroQWERTY platform.

To assure a comparable amount of typing activity between the in-clinic and at-home settings, only subjects who aggregated at least 15 min of typing data during their corresponding at-home baseline period were included in the analysis. Though, importantly, data at home were sparsely distributed over the multi-day baseline period, whereas the in-clinic data were concentrated in a 15-min continuous typing task. To manage this sparsity in the at-home data, we applied the concept of valid window to filter typing gaps and low-activity intervals. A valid window was defined as a data sequence of at least 30 keystrokes within a 90-s time interval. We excluded 5 PwP and 3 control subjects from the analysis because they did not reach the equivalent 15-min active typing threshold (10 valid windows) during the baseline period.

**Figure 1:**
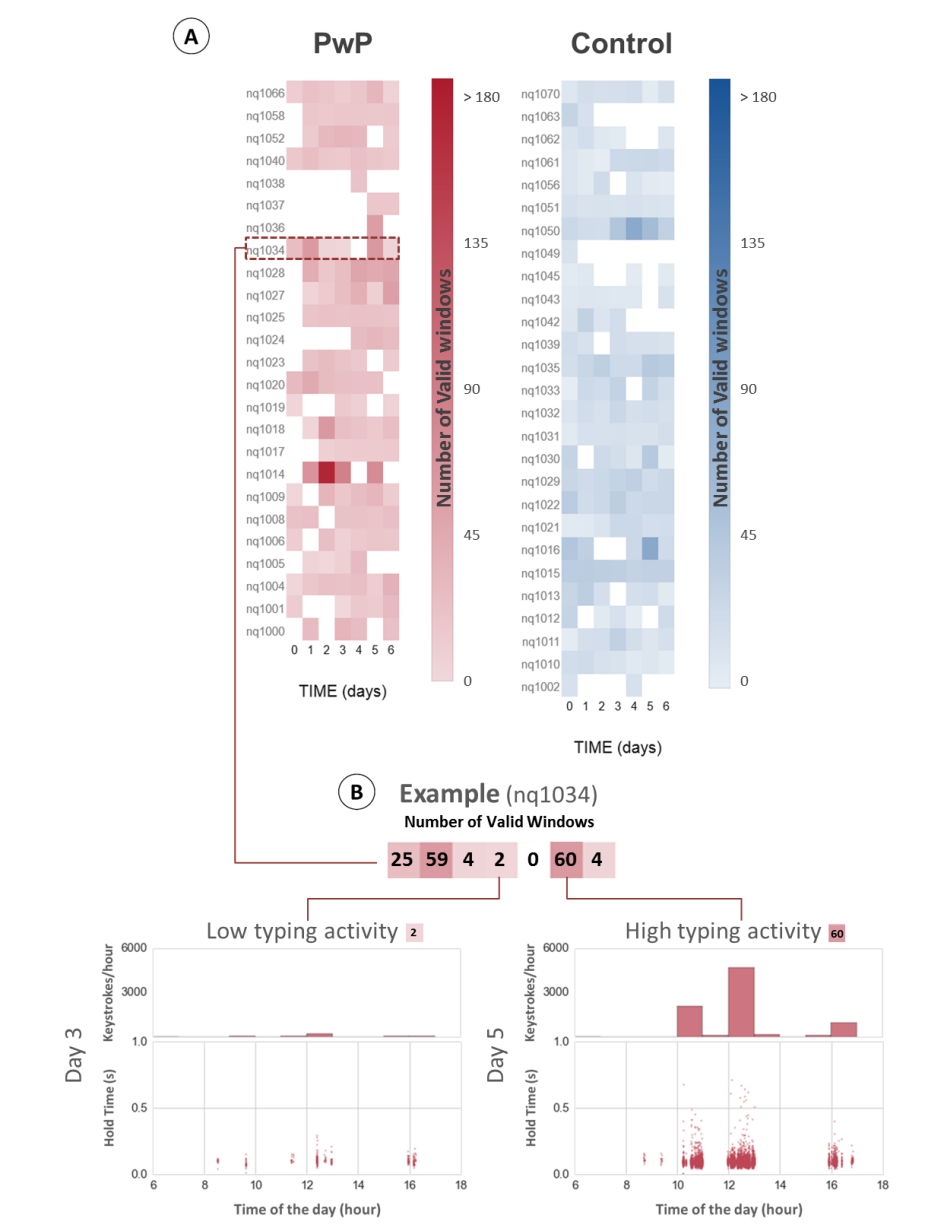
At-home typing activity. Panel A represents the amount of typing data collected from each of the 52 subjects (25 PwP, 27 CNT) included in the analysis. The red (PwP) and blue (CNT) color scales indicate daily typing activity measured as the number of valid typing windows provided by each subject during the analysis period. We defined a valid window as a sequence of at least 30 keystrokes within 90 s. Panel B illustrates the variability in the amount of typing data with an example from a single PwP subject.

A summary of demographic and clinical information for the resulting cohort, 25 PwP and 27 healthy controls, can be found in Table 1. Regarding PD severity, all PwP subjects were newly diagnosed cases and in the very early stages of the disease, with a mean UPDRS-III score of 20.48 points. For reference, a score of 20 points is typical of patients with very mild disease severity [13]. The 2 groups were matched in age, gender, and volume of daily typing. A detailed representation of the at-home baseline data collected for each subject is shown in Figure 1. The plot illustrates the heterogeneity of subjects’ typing behaviors, which we previously identified as one of the potential risks for the validation of our approach in a natural at-home setting. Participants typed an average of 24.07 (SD 15.13) min per day, with 2.79 min per day for the less-active subject and 83.14 min per day for the most active subject. This variability was also observed within subjects’ typing routines, as several participants did not present a consistent typing activity over the monitored time period. These characteristics in the at-home spontaneous typing data contrast with the quasi-continuous signal captured during the in-clinic typing test. In the *Analysis* subsection, we will explain how we addressed these differences to allow us to compare the performance of the algorithm for the in-clinic and at-home scenarios.

**Table 1:** Comparison of the clinical and demographic variables between the Parkinson's disease and control groups. From the total participants, 52 provided a sufficient amount of at-home typing data (a cumulative total of at least 15 min). The UPDRS-III scale ranges from 0 to 108 (a higher score indicates more severe impairment and disability). For reference, a score of 20 points is typical of patients with very mild disease severity.

Variable	People with Parkinson’s (n=25)	Healthy controls (n=27)	*P* value
UPDRS-III^a^, mean (SD)	20.48 (6.56)	1.93 (1.84)	<.001
Age in years, mean (SD)	60.2 (12.0)	60.81 (10.63)	.73
Number of women, n (%)	12 (48)	14 (52)	.79
Number of men, n (%)	13 (52)	13 (48)	.79
Daily typing in minutes, mean (SD)	24.58 (15.91)	23.58 (14.68)	.61

^a^UPDRS-III: Unified Parkinson’s Disease Rating Scale (Part III).

### Data-Collection Platform

The neuroQWERTY platform provides functionality for user registration and log-in, distribution of the data-collection software, and storage and management of the typing data. Once installed, the data-collection software runs in the background, capturing the timing information of any keyboard input. More specifically, for each keystroke, the program stores the timestamps corresponding to the press and release events. To ensure privacy, the collected information did not include the content of each specific key. However, each keystroke was labeled with its corresponding key category; special key, right side key, or left side key, to allow filtering of key types that engage nonstandard digit kinematics (eg, SHIFT). The mean for measured temporal resolution of the data-collection software was 3 (SD 0.28) msec.

The typing information, linked to each user account, was automatically sent to a remote server for analysis. Privacy and data security were assured at 3 levels: at the client level, the data transmission level, and the data storage level. Any typing data stored on the local machine (which again, did not include the content of the keys) were encrypted and deleted from the device after sending to the remote server. Data transmission was protected through secure hypertext transfer protocol. At the server level, data were stored in the database in an encrypted format and were only accessible by authorized database administrators or by the user himself after authentication.

**Figure 2:**
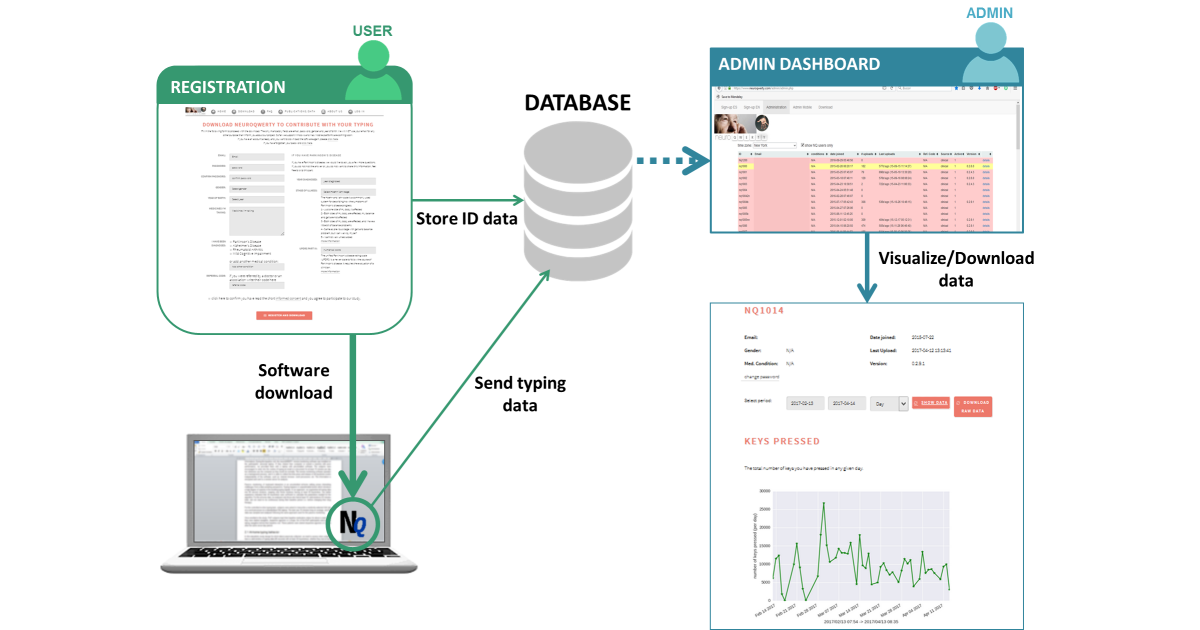
The neuroQWERTY platform. This platform was designed to allow for automatic data retrieval of typing data collected at home and remote management by a study coordinator. Operationally, an account in the neuroQWERTY platform was created for each participant in the study. The data-collection software was downloaded and installed in their users’ personal laptop to enable remote data collection. The data, linked to each user account, was encrypted and automatically sent to a remote server through their home Internet connection. The neuroQWERTY platform also implemented an administrator module to provide the study coordinators with an interface to control and visualize participants’ typing activity.

Finally, the platform included an administrator module to provide the study coordinators with an interface to access and control participants’ typing activity. The administrator dashboard implemented a color code to alert study coordinators about users’ prolonged inactivity. Web-based visualization of the subjects’ typing data was also enabled, including the daily key count and the temporal representation of the raw key typing dynamics. A schema of the complete neuroQWERTY platform framework is shown in Figure 2.

### Analysis

We evaluate the classification ability of the neuroQWERTY index (nQi) to separate a group of healthy controls from an early PD population using the typing data collected during subjects’ natural interaction with their laptop. The nQi is the output of a computational algorithm that uses the information contained in the sequences of hold times, the time between pressing and releasing each key on a mechanical keyboard, to detect evidence of PD motor impairment. This algorithm was first introduced in Giancardo et al [12], where we showed its ability to accurately discriminate early PwP from healthy controls by analyzing the data collected in a controlled in-clinic typing task.

**Figure 3:**
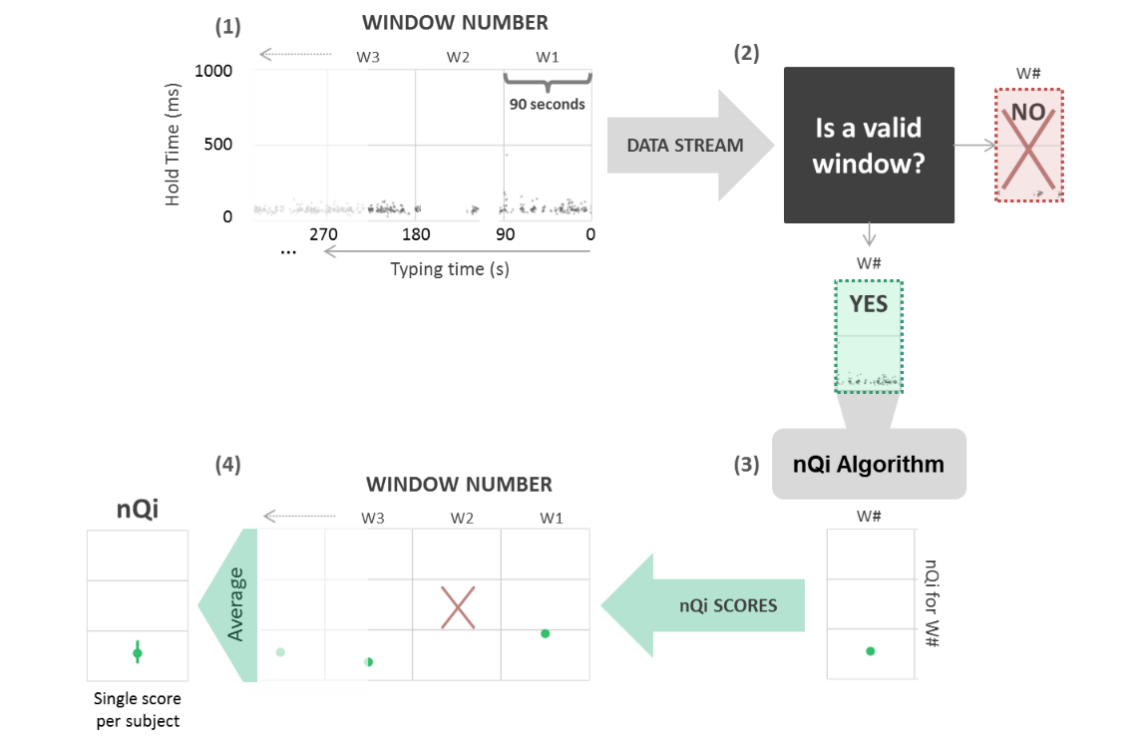
Algorithm pipeline. The figure represents the pipeline to generate a single neuroQWERTY index (nQi) from a stream of typing data. (1) The typing signal is defined as the time series of hold times corresponding to each keystroke within a typing routine. This signal is split by nonoverlapping 90-s windows that the algorithm will evaluate as independent typing units. (2) Only windows with at least 30 keystrokes within the 90-s interval are analyzed. (3) The neuroQWERTY algorithm, previously trained on a separate in-clinic dataset, computes a single numerical score from each independent window. (4) The final nQi is computed as the average of the window-level scores.

A representation of the algorithm pipeline is shown in Figure 3. The hold time signal is split into 90-s windows that are analyzed as independent typing units. Applying variance analysis, the information within each unit is reduced to a 7D feature vector that is used as the input of an ensemble model consisting of a family of linear support vector regressors (SVR). An independent window-level score is calculated as the median of the outputs of each linear SVR. Finally, the final nQi score is computed as the mean of the window-level scores. The feature analysis and algorithm parameter estimation are described in detail in our previous paper, Giancardo et al [12].

The ensemble linear-SVR model was trained using an external dataset that included the typing signals of 18 early-PD subjects and 13 healthy controls different than the ones included in this study. This training set was fully collected in a controlled in-clinic environment, that is, during a timed copy task integrated into the study clinical visit. Therefore, the main question we try to answer in this work is whether our algorithm can generalize to typing data acquired in a fully uncontrolled home-based scenario where the subjects are free to use their laptops as they normally do.

**Figure 4:**
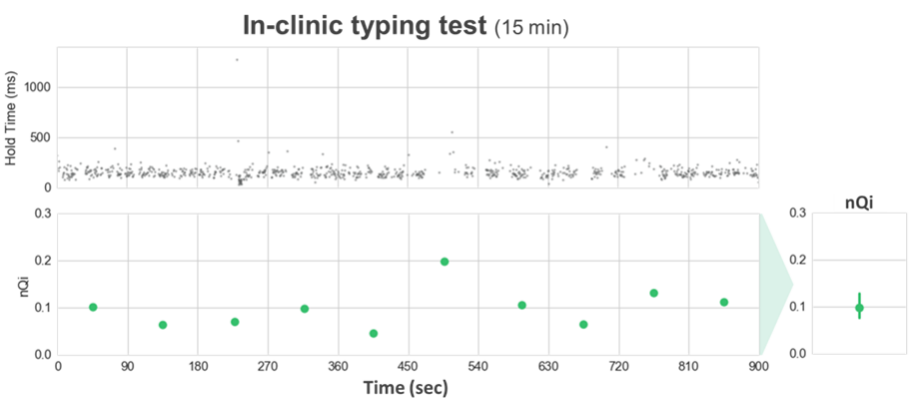
Example of the application of the neuroQWERTY algorithm in an in-clinic typing test.

**Figure 5:**
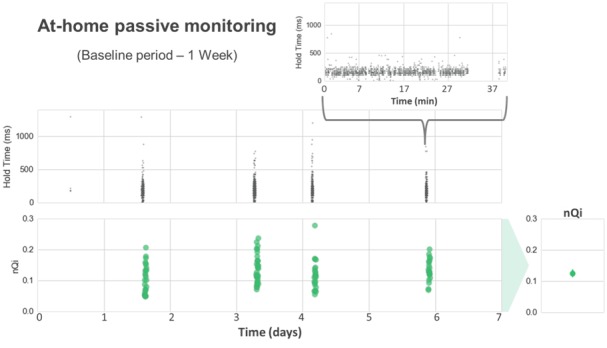
Example of the application of the neuroQWERTY algorithm in the at-home setting. The neuroQWERTY algorithm described in Figure 3 can be used indistinctly to evaluate controlled or natural typing data. This figure represents the at-home typing data and corresponding scores for the same subject shown in Figure 4 (note different time scales used in Figure 4 and Figure 5). Although the uncontrolled activity appears in unpredictable bursts that introduce a high degree of sparsity, our window-based approach allows to analyze the at-home data using the same method applied for the quasi-continuous in-clinic data.

In Figures 4 and 5, we present an example of the application of the algorithm in a controlled in-clinic task opposed to the same process in an at-home typing setting. The comparison of the hold time data representation between in-clinic (panel A) and at-home (panel B) illustrates the sparsity introduced in the typing signals by the uncontrolled at-home environment, where the keyboard is only used intermittently as opposed to the continuous stream of data collected in the controlled in-clinic setting. To account for the sparsity of the hold time series, only valid windows, subsequences of at least 30 keystrokes within each 90-s interval, are included in the analysis. Special key types (eg, SHIFT) that may engage nonstandard digit kinematics are excluded from the hold time (HT) data collection. Due to the duration of the at-home baseline period, the volume of data collected at home is generally greater than the in-clinic data available for each subject. We measured an average of 9.62 (2.13) valid windows per subject during the in-clinic typing test and collected an average of 112.33 (70.65) valid windows per subject from the 7-day at-home typing activity. The extended at-home monitoring period increases subjects’ at-home sample size, which tends to reduce the individual’s internal variance intensified in this uncontrolled environment.

The data analysis comprised 2 phases. First, we evaluated the influence of the controlled typing task in subjects’ normal typing behaviors. Specifically, we compared the measured values of the raw typing metrics, flight time (FT, delay between consecutive key presses), and HT (time between pressing and releasing a key), and the computed nQi scores between the in-clinic and at-home typing settings. To assess the similarity in the relationship between the in-clinic and at-home metrics, we computed the line of best fit and correlation coefficient. We completed this first part of the analysis with a Bland-Altman plot [14] to evaluate the nQi score’s agreement between the 2 typing settings.

In the second part of the analysis, we assessed the classification performance of the neuroQWERTY method using the at-home typing data and compared these results with the ones obtained in the clinic. The results obtained in each typing settings were evaluated using the following metrics: receiver operating characteristic (ROC) analysis and the Mann-Whitney *U* test to reject the null hypothesis that the healthy controls and the Parkinson’s samples come from the same distribution. For the ROC analysis, we used a sampling with replacement method to define a distribution of curves from which we computed the average area under the ROC curve (AUC) and its CIs. Each curve is built on an iterative process that monotonically increases the value of the index to define a dynamic threshold. On each iteration, a sensitivity/specificity pair is computed using the current threshold value. These pairs are used to draw the resulting ROC curve. The value of the AUC can be interpreted as the probability of the classifier to rank a randomly chosen positive instance higher than a randomly chosen negative one [15]. To evaluate the equivalence of our method between the in-clinic and at-home settings, we estimated the percentage agreement and the statistical difference of the resulting ROC curves (DeLong test [15]).

## Results

**Figure 6:**
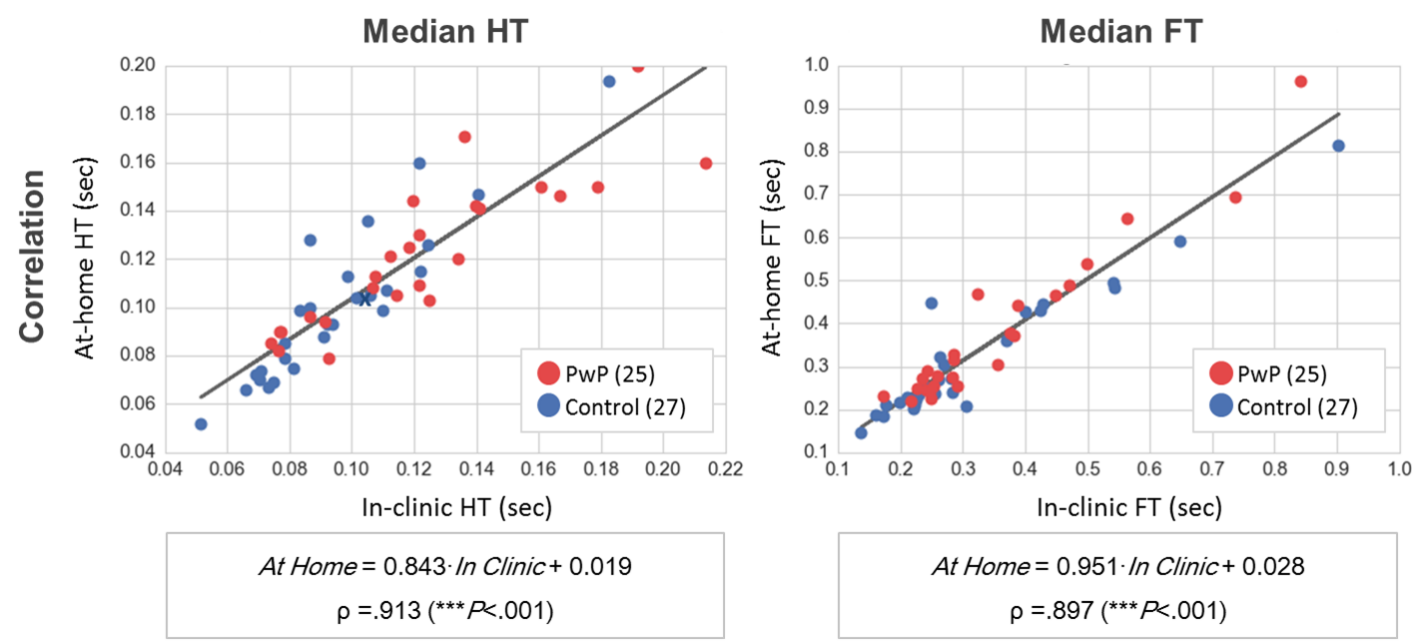
Comparison of raw typing metrics between in-clinic and at-home typing settings. The figure shows the correlation of the raw typing metrics, hold time (HT; time between pressing and releasing a key), and flight time (FT; delay between two consecutive key presses), between in-clinic and at-home settings. Each point represents the metric coordinates (in-clinic, at-home) for each of the 52 participants included in the analysis. Both HT and FT values are very similar independently of the typing scenario, as shown by the correlation coefficient values. These results suggest that the in-clinic task does not alter the way subjects type in comparison with their natural typing at-home, which supports our hypothesis that the neuroQWERTY algorithm, built in an in-clinic setting, could be applied to evaluate motor impairment using the typing data from an uncontrolled at-home setting.

The results of the raw typing variables agreement between in-clinic and at-home are shown in Figure 6. We evaluate the statistical relationship, line of best fit, and correlation for the median flight and HT measured in-clinic and at-home settings. The values of the 2 typing metrics are very similar independently of the typing scenario, as shown by correlation coefficient values, .913 for the median FT and .897 for the median HT, and also by the slope of the computed line of best fit, close to 1 in both cases.

**Figure 7:**
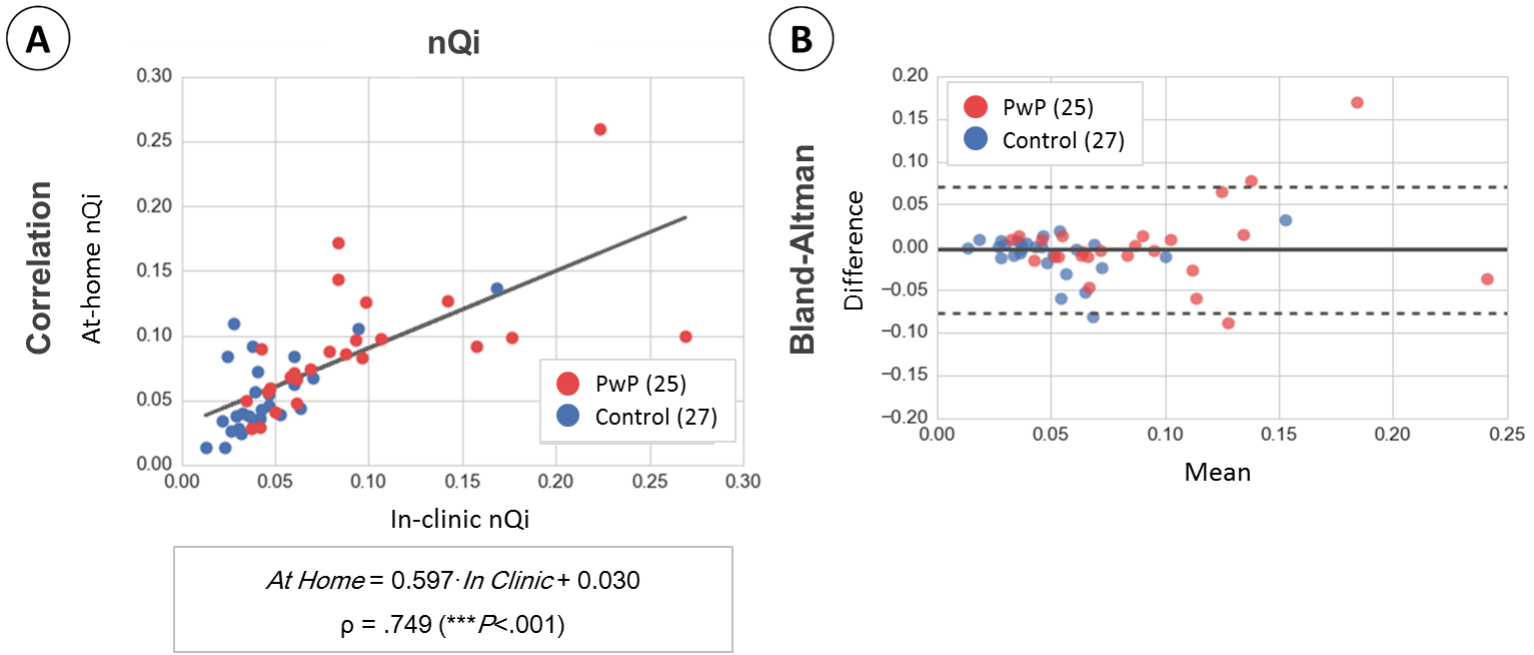
Comparison of neuroQWERTY index (nQi) between in-clinic and at-home typing settings. We evaluated the influence of the typing setting in the nQi scores by applying a similar analysis as described in Figure 6 for the raw typing metrics. Panel A shows the correlation of the nQi scores computed in-clinic and at-home. Panel B includes the results of the Bland-Altman analysis to evaluate the agreement of our method in the two typing scenarios. The black line shows the mean difference (d) and the top and bottom dashed lines show the limits of agreement (LoA, d±1.96×SDd).

A similar analysis applied to the nQi scores is shown in Figure 7. The linearity between the in-clinic and at-home settings for this variable is weaker than that observed on the raw typing variables. The correlation coefficient is .749 in this case, and the slope of the line of best fit is not as close to the unit, .597. However, the agreement analysis suggests a correspondence between the scores measured in-clinic and at-home settings, with a 92% (48/52) of the cases falling between the Bland-Altman limits of agreement (LoA).

**Figure 8:**
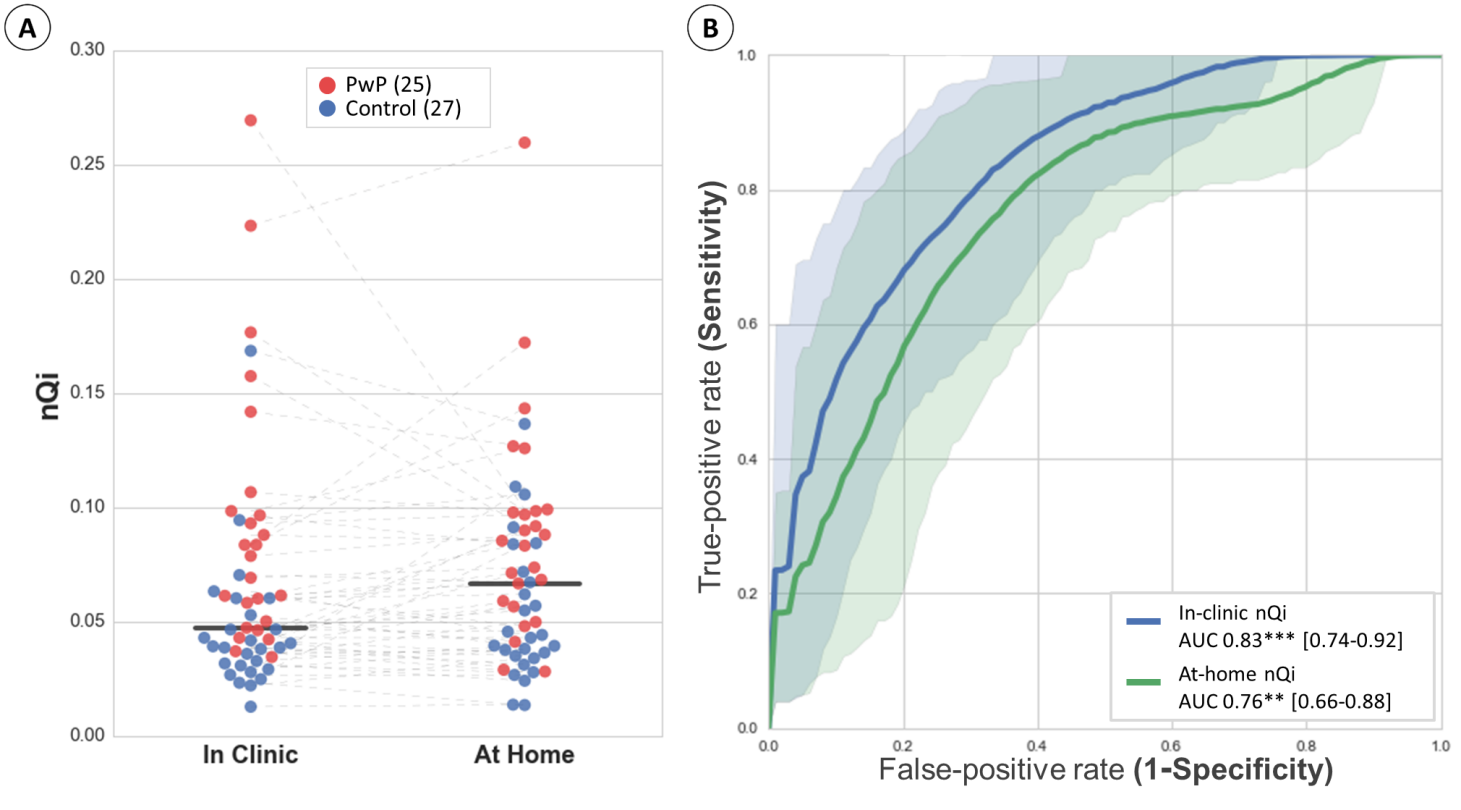
Comparison of neuroQWERTY index (nQi) performance between in-clinic and at-home typing settings. Panel A scatterplot illustrates the in-clinic and at-home nQi scores in a patient level. The two black lines represent the classification thresholds computed in-clinic (nQi=0.0473) and at-home (nQi=0.0667). These thresholds were estimated for closest-to-(0,1) cutoff points that maximize sensitivity/specificity pairs. Panel B presents the comparison of the receiver operating characteristic (ROC) curves showing the classification rate for the in-clinic and at-home nQi. The plotted curves are the average result of the bootstrapped ROC analysis and the shadowed areas represent the corresponding CIs [5th-95th]. The statistical significance of the Mann-Whitney U test is estimated to reject the null hypothesis that the two groups, PwP and CNT, come from the same population. It is noted as: P<.001(***), P<.01(**), and P<.05(*).

In terms of classification performance, the nQi worked well with the at-home typing data (Figure 8 and Table 2). The absolute nQi scores tended to be larger for at-home data relative to the corresponding in-clinic values (Figures 7 and 8), but in both cases the scores for PwP were generally greater than for healthy controls. The similarity in classification performance for in-clinic versus at-home data can also be seen by comparing the ROC curves (Figure 8 and Table 2). The cutoff point was estimated using the closest-to-(0,1), that is, the use case that maximizes the sensitivity/specificity pair [16] (Table 2). The neuroQWERTY algorithm discriminates our early PD population from healthy controls with an AUC of 0.76 (0.66-0.88) using the typing data from the at-home natural interaction on a mechanical keyboard. In the clinic, the results of the analysis, a controlled typing task in the same cohort, achieved an AUC of 0.83 (0.74-0.92). According to the DeLong test, the ROC AUC difference between in-clinic and at-home settings was not significant (*P*=.18). The percentage agreement of the results of our method between the 2 typing settings was 79%.

**Table 2:** The neuroQWERTY index (nQi) performance comparison. The classification performance achieved at-Home is comparable with the results obtained in a controlled in-clinic. The statistical significance is computed with 2-sided Mann-Whitney *U* test to reject the null hypothesis that PwP and healthy control subjects come from the same population.

Metric	nQi^a^ Score	
	In-clinic	At-home
Mean (SD) for PwP^b^ (n=25)	0.092 (0.058)	0.090 (0.048)
Mean (SD) for healthy controls (n=27)	0.046 (0.029)	0.054 (0.030)
AUC^c^ (5th-95th)	0.83 (0.74-0.92)	0.76 (0.66-0.88)
Significance	*P*<.001	*P*<.01
Sensitivity/specificity	0.77/0.72	0.73/0.69
DeLong test	*P*=.18	*P*=.18
Percentage agreement	79%	79%

^a^nQi: neuroQWERTY index.

^b^PwP: people with Parkinson’s.

^c^AUC: area under the curve.

## Discussion

### Principal Findings

The results of this study represent a step toward a transparent and ubiquitous motor sign assessment tool for PD. In our previous work [12], we introduced the neuroQWERTY method, a machine learning algorithm trained to quantify PD severity through the analysis of the typing patterns found in the time series of HT. Our method was able to discriminate an early PD population from a matched control group using the typing data collected during a controlled in-clinic task. In this paper, we tested the validity of our algorithm in an uncontrolled at-home setting. The neuroQWERTY platform allowed us to unobtrusively collect the typing information from a cohort comprising 30 PwP and 30 matched healthy controls. Most (52/60, 90%) of the study subjects (25 PwP, 27 healthy controls) provided enough data during the follow-up period to evaluate the nQi at home. Our neuroQWERTY algorithm, built using a separate in-clinic dataset, was able to distinguish PwP from healthy controls through the analysis of natural at-home typing patterns with an AUC of 0.76 and 0.73/0.69 sensitivity/specificity. Despite the sparsity and heterogeneity introduced by each subject’s routine use of the computer, the neuroQWERTY method performed nearly as well in the at-home setting as it did when applied in a controlled in-clinic typing task (AUC 0.83 and 0.77/0.72 sensitivity/specificity). The nQi scores presented no significant differences between the de-novo PwP group (19) and the subset of PwP participants on medication (Multimedia Appendix 1).

The comparison of the raw typing metrics between the 2 typing scenarios suggests that the in-clinic typing test does not affect the way people type with regard to their normal use of the computer at-home. The correlation coefficient for the median HT between in-clinic and at-home was ρ=.913 (*P*<.001). A similar analysis applied to the resulting nQi shows a weaker correlation between the scores computed from the in-clinic and at-home typing data (ρ=.749, *P*<.001, with 48 out of 52 or 92% of the samples within the Bland-Altman LoA). This could be due to the sensitivity of the algorithm to small changes in the HT values between the 2 typing settings. Despite the weaker correlation, the classification performance of the neuroQWERTY method applied at-home was similar to the classification performance in-clinic (statistically indistinguishable by the DeLong test: *P*=.18, percentage agreement: 79%).

These results support our initial hypothesis that PD-related motor signs affect the way patients interact with mechanical keyboards and are, therefore, detectable through the analysis of their regular typing patterns. The ability of the neuroQWERTY algorithm to extrapolate the patterns learned from a separate in-clinic dataset to correctly identify PD-characteristics in the at-home typing data provides external validity to our method. Being able to generalize to data collected from the hardest possible scenario can also be seen as an opportunity to improve these results by implementing an at-home-specific algorithm that, trained on passively collected data, will be able to identify the useful information and learn to filter the several different sources of noise introduced by the uncontrolled at-home setting. Although the current clinical standard, UPDRS, outperforms our technique, the goal of neuroQWERTY is not to replace UPDRS but to provide a method that enables PD assessment when a clinician is not available. Nevertheless, it would be interesting to explore the potential for the nQi approach to provide a meaningful indication of UPDRS. Despite being based just on a distal upper limb movement, with our limited dataset we did find a significant moderate correlation between the nQi scores and UPDRS-III (Multimedia Appendix 2).

### Main Contribution and Limitations

Using the timing information from users’ natural typing activity provides our approach with a number of advantages over alternative solutions, but it also poses some limitations. An obvious concern is the level of compliance, since the method depends on sufficient use of the computer. In the study cohort, a high percentage of the participants (90%) provided enough data during the 7-day follow-up to shape a representative typing pattern. Our user adherence results highlight the advantages of passive data collection in contrast with other existing active task-based methods. Task-based methods are commonly limited by their dependence on users' active engagement to collect information through a series of standardized tasks, which introduces potential artifacts due to subject-awareness of being monitored [17] and hinders user compliance. As an example, in the context of the mPower study less than 10% of the participants provided 5 or more finger-tapping data points over a 6-month follow-up period [10].

Although our passive data-collection approach significantly increases user adherence, some strategies could be employed to maximize it. A possible solution to reduce the rate of excluded participants would be collecting data not only from laptop use but from any electronic device that entails typing. In Arroyo et al [18], it was proven that a similar approach can be used to detect PD via smartphone touchscreen typing. Integrating data from multiple devices would provide a more continuous stream of data; therefore, a deeper insight to assess PD signs.

### Future Work

Proving that our method can distinguish an early PwP cohort, with an average years from diagnosis of 1.66 (1.20) and mean UPDRS-III score of 20.48 (6.56) from a matched healthy control group is an indicator that at-home typing patterns can capture PD-specific motor characteristics that are mild in this stage of the disease. This could have an impact in early detection of PD as machine learning algorithms can be trained to detect very subtle variations in the input data, in this case changes in the typing patterns, caused by early motor manifestations of PD that may often go unnoticed by clinicians [19]. The neuroQWERTY software could be installed on PD-risk populations’ devices to enable earlier diagnosis, when putative neuroprotective treatments could stop neurodegeneration. Clinical studies in an as-yet-undiagnosed population would be needed to validate the sensitivity and applicability of our tool for this specific use case.

Although our classification results show promise, our longer term goal is to develop a tool to objectively track progression of PD signs. This would provide clinicians with invaluable information to tailor treatments to patients’ specific conditions. Today, there is no known cure for PD, but available medications can help manage its symptoms. Individualized treatment regimens are crucial to provide optimized symptom control [20]. Medications adjustments rely mainly on the information gathered by movement disorder experts during clinical visits. This limits decision making to subjective follow-up examinations scheduled every 2 to 6 months. Ideally, our approach could be applied not only to classify but also to track PD progression and therapeutic efficacy. This would require further validation in a longitudinal study to evaluate the precision of the neuroQWERTY approach to monitor PD progression over time.

### Conclusions

Relying on the analysis of the temporal patterns from the daily interaction with electronic devices, our approach introduces a new way to objectively and unobtrusively detect motor impairment in PD, providing access to quasi-continuous ambulatory data without harming user compliance. The main purpose of this analysis was to evaluate the validity of the nQi, an in-clinic-built digital marker for early PD motor impairment, in an uncontrolled at-home setting. The classification performance of the algorithm was statistically similar in its ability to discriminate 25 PwP and 27 healthy controls from the at-home typing data (AUC of 0.76 and 0.73/0.69 sensitivity/specificity) nearly as well as it was able to separate them using the in-clinic typing patterns (AUC 0.83 and 0.77/0.72 sensitivity/specificity). These results prove that the data collected from subjects’ routine use of the computer are also valid to detect PD-related motor signs, getting us closer to our ultimate goal of providing an objective ambulatory tool to monitor PD progression.

## Appendix

Multimedia Appendix 1 Influence of medication in nQi assessment. Six of the PwP study participants were on rasagiline when they joined the study. Panel A shows no significant differences between the nQi scores of the PwP participants using rasagiline and the de-novo group. Panel B shows the UPDRS-III scores for the CNT, PwP De-Novo and PwP On-Medication groups. Participants on rasagiline scored higher up in the motor scale in comparison with the average score in the full PwP cohort. It is possible that this greater severity in their baseline status could be masking the effect of medication.

Multimedia Appendix 2 nQi correlation with motor clinical standard. The figure shows the correlation between UPDRS-III scale and the nQi scores measured in-clinic (Panel A) and at-home (Panel B). Correlations were significant in both typing settings and moderate as shown by the correlation coefficients; .50 in-clinic and .34 at-home. Despite being based just on distal upper limb movement, with our limited dataset we did find a significant moderate correlation between the nQi scores and UPDRS-III.

## Abbreviations

AUC: area under the ROC curve
HT: hold time
LoA: limits of agreement
nQi: neuroQWERTY index
PD: Parkinson’s disease
PwP: People with Parkinson’s
ROC: receiver operating characteristic
SVR: support vector regressor
UPDRS-III: Unified Parkinson's disease rating scale, Part III
