# Supplementary figures and images for: Detecting Motor Impairment in Early Parkinson’s Disease via Natural Typing Interaction With Keyboards: Validation of the neuroQWERTY Approach in an Uncontrolled At-Home Setting
Source: J Med Internet Res. 2018 Mar 26;20(3):e89. doi: 10.2196/jmir.9462 (PMC5891671; doi:10.2196/jmir.9462)

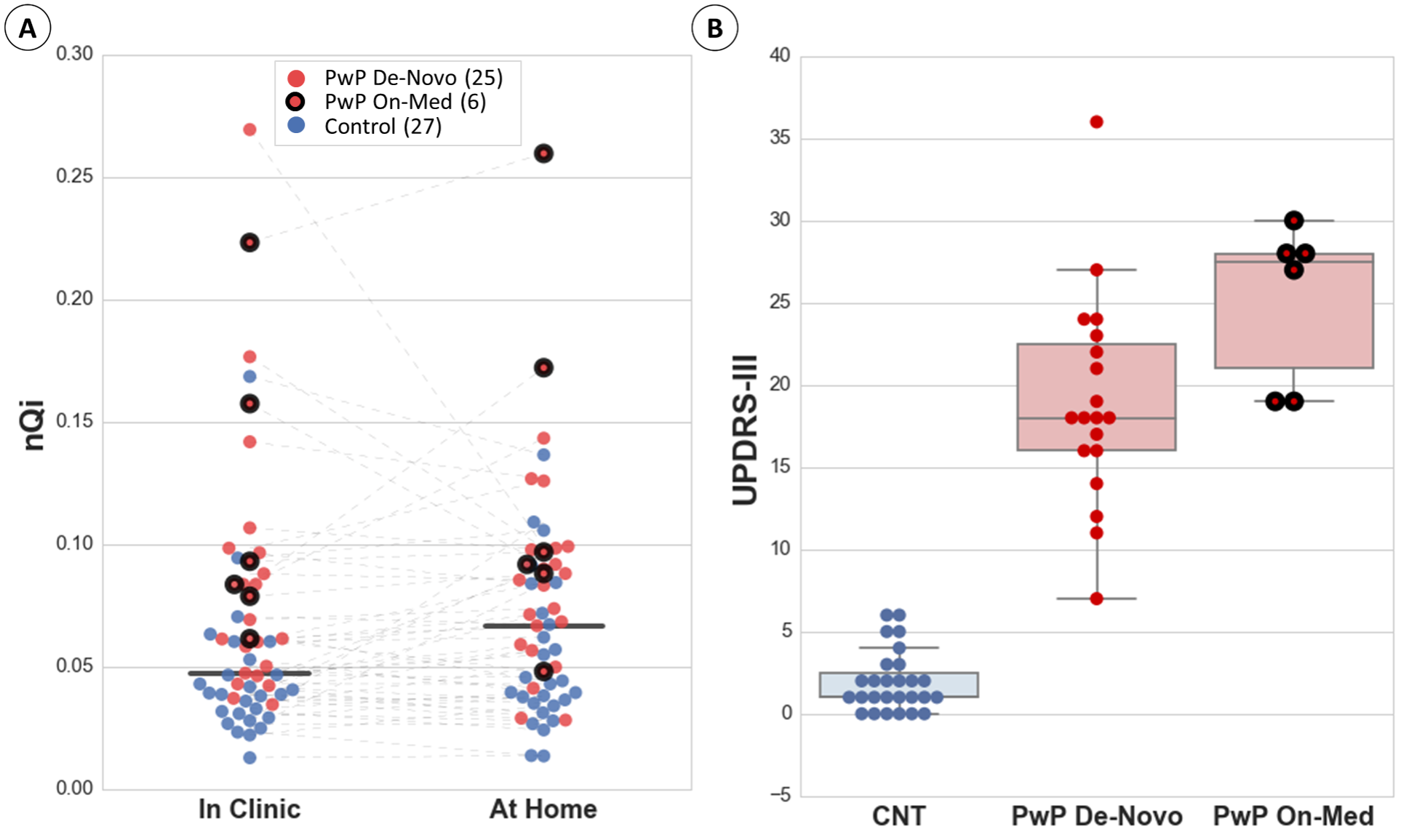

Supplement: Multimedia Appendix 1 [file jmir_v20i3e89_app1.png]

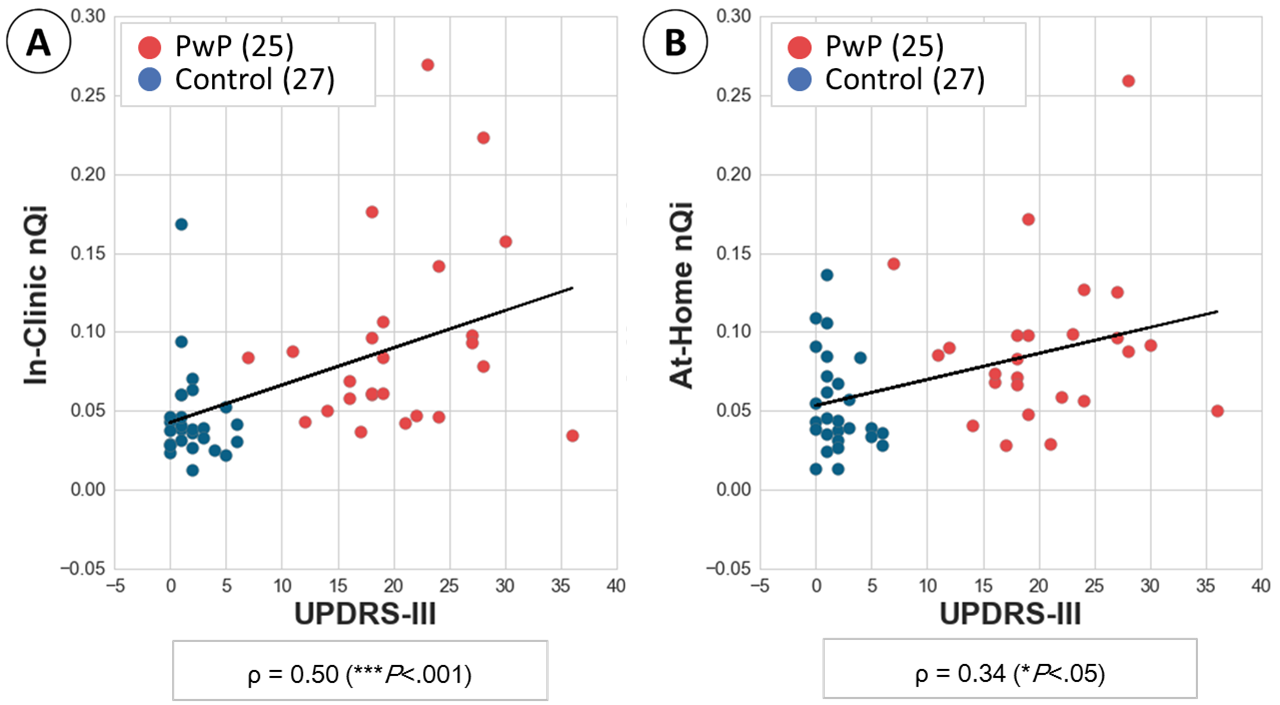

Supplement: Multimedia Appendix 2 [file jmir_v20i3e89_app2.png]
